# Initial-Stage Primary Intraosseous Squamous Cell Carcinoma Derived from Odontogenic Keratocyst with Unusual Keratoameloblastomatous Change of the Maxilla: A Case Report and Literature Discussion

**DOI:** 10.1155/2018/7959230

**Published:** 2018-04-19

**Authors:** Kentaro Kikuchi, Fumio Ide, Shota Takizawa, Seiji Suzuki, Hideaki Sakashita, Tie-Jun Li, Kaoru Kusama

**Affiliations:** ^1^Division of Pathology, Department of Diagnostic and Therapeutic Sciences, Meikai University School of Dentistry, 1-1 Keyakidai, Sakado, Saitama 350-0283, Japan; ^2^Second Division of Oral and Maxillofacial Surgery, Department of Diagnostic and Therapeutic Sciences, Meikai University School of Dentistry, 1-1 Keyakidai, Sakado, Saitama 350-0283, Japan; ^3^Department of Oral Pathology, Peking University School of Stomatology, 22 South Zhongguancun Avenue, Haidian District, Beijing 100081, China

## Abstract

Primary intraosseous squamous cell carcinoma (PIOSCC) is a rare malignant neoplasm derived from odontogenic epithelial remnants in the central jaw bone. Most PIOSCCs originate from odontogenic cysts with a nonkeratinized epithelial lining, especially from radicular/residual and dentigerous cysts. There have been few reports of PIOSCCs derived from the odontogenic keratocyst (OKC), particularly those describing pathological features at the initial stage. The diagnosis of PIOSCC is difficult and based on exclusion of other carcinomas, including metastatic tumors from other primary sites. Here, we report an extremely rare case of initial-stage PIOSCC derived from the OKC with unusual keratoameloblastomatous change of the maxilla.

## 1. Introduction

Primary intraosseous squamous cell carcinoma (PIOSCC) is a carcinoma arising from the central bone without any initial connection to various epithelia [1]. In 2017, in the World Health Organization (WHO) classification of tumors, the name was changed from PIOSCC to primary intraosseous carcinoma (PIOC), not otherwise specified (NOS) [2]. The diagnosis of PIOSCC requires specific criteria to be met, including the absence of oral ulceration or communication with the overlying mucosa, the absence of a distant primary tumor at the time of diagnosis, and histologic evidence of squamous cell carcinoma [1, 2]. The diagnosis of PIOSCC can be difficult, and it must be differentiated from other malignancies such as ameloblastic carcinoma and metastatic carcinomas; when it causes destruction of the cortical bone and invades adjacent soft tissues, it may be confused with a carcinoma of the oral mucosa. Most PIOSCCs originate from the epithelial lining of odontogenic cysts, especially radicular, residual, and dentigerous cysts [1]. There are about 120 reported cases of PIOSCCs arising from cysts, with 25 of them being derived from odontogenic keratocysts (OKCs) [3, 4]. PIOSCCs arising from OKCs are extremely rare, and fewer than 30 have been reported so far. Few reports have presented clear evidence of histological transition between squamous cell carcinoma and the odontogenic epithelial lining of the cyst wall. Here, we present a very rare case of initial-stage PIOSCC derived from the OKC with unusual keratoameloblastomatous change of the left maxilla.

## 2. Case Presentation

**Figure 1:**
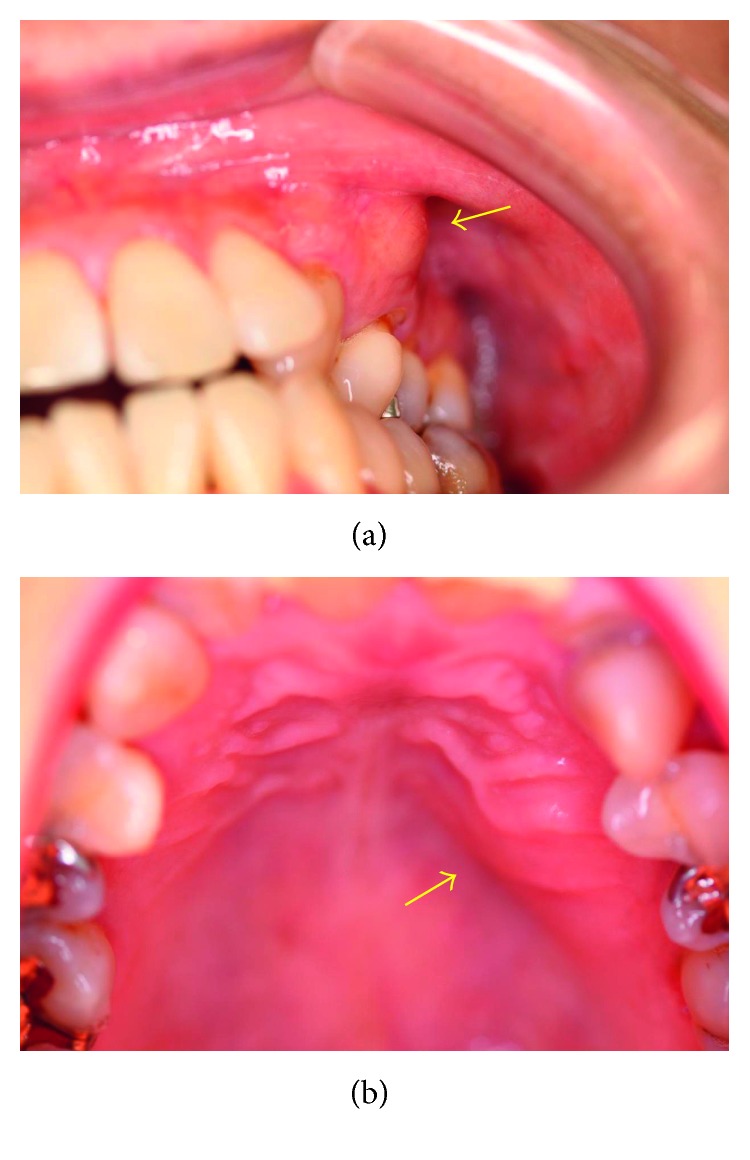
Intraoral appearance of the upper jaw. (a, b) Swollen nonulcerative mucosa in the buccal and palatal side gingiva (yellow arrow).

**Figure 2:**
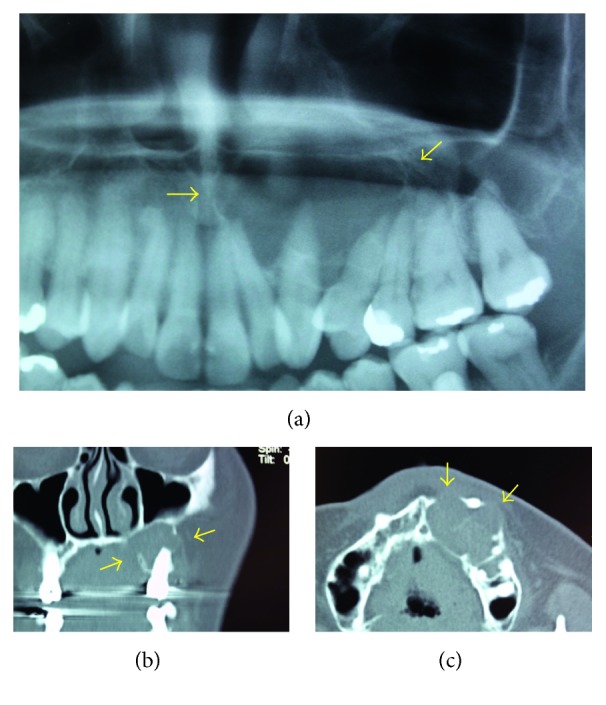
Radiologic and computed tomography (CT) findings. (a) Panoramic radiograph showing a well-delineated radiolucent lesion (yellow arrows). (b, c) CT showing expansion and cortical bone osteolysis (yellow arrows).

**Figure 3:**
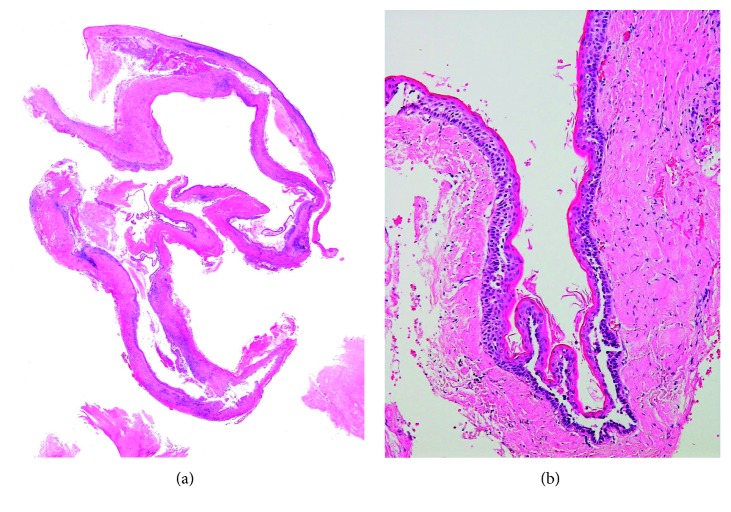
Microscopic features of the biopsy specimen. (a) Survey view of the biopsy specimen. (b) The cyst wall lined by a folded, thin, regular parakeratinized epithelium without rete ridges, and the basal layer lined by palisaded columnar cells with hyperchromatic nuclei (HE; original magnification: ×12.5 (a) and ×100 (b)).

**Figure 4:**
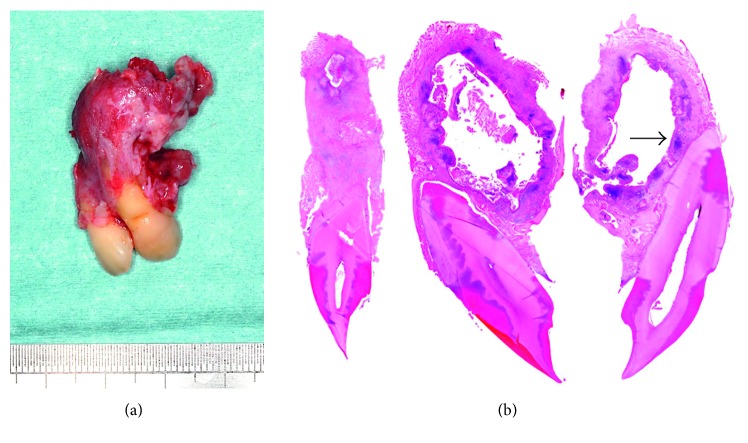
Macroscopic and survey view of the surgically resected specimen. (a) Surgical specimen. (b) Invasive atypical squamous cell nests in the cyst walls (black arrow) (HE; original magnification: ×1 (b)).

**Figure 5:**
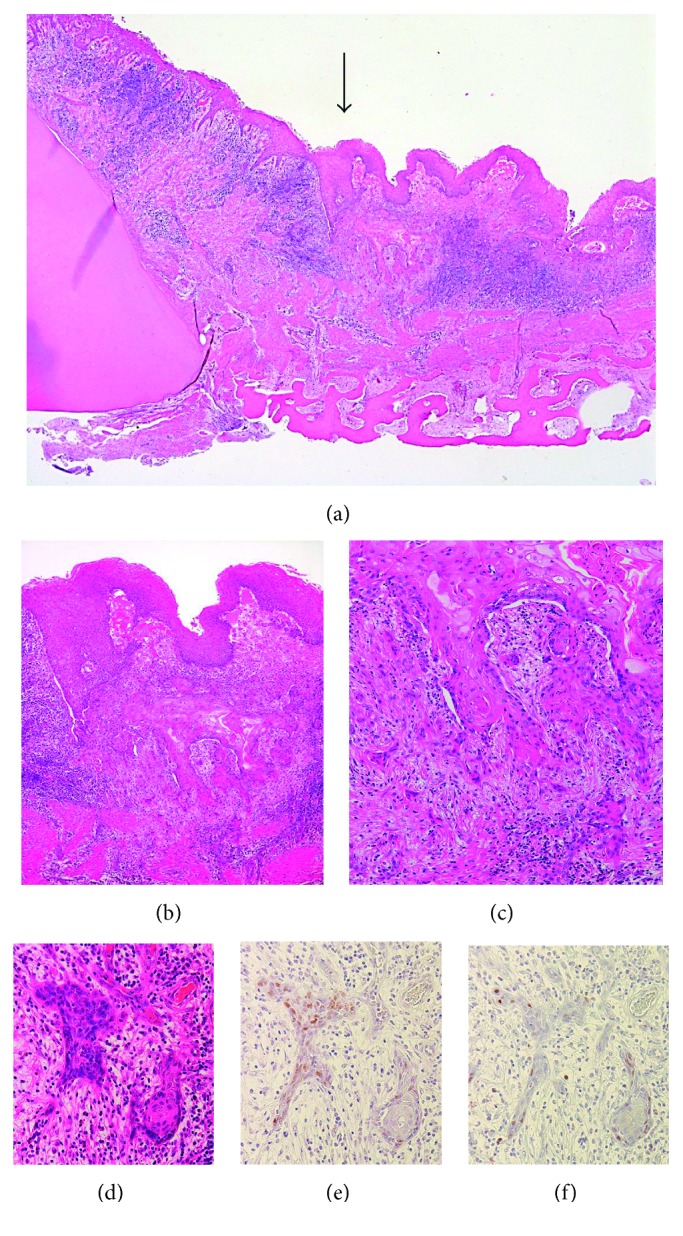
Microscopic features of the surgical specimen. (a) Invasion of atypical epithelium (black arrow). (b) Invasive squamous epithelium irregularly migrating from the epithelial cyst lining similar to SCC. (c) High-power magnification. (d) Invasive nests, (e) p53, and (f) Ki-67 (HE and IHC; original magnification: ×12.5 (a), ×40 (b), ×100 (c), and ×200 (d), (e), and (f)).

**Figure 6:**
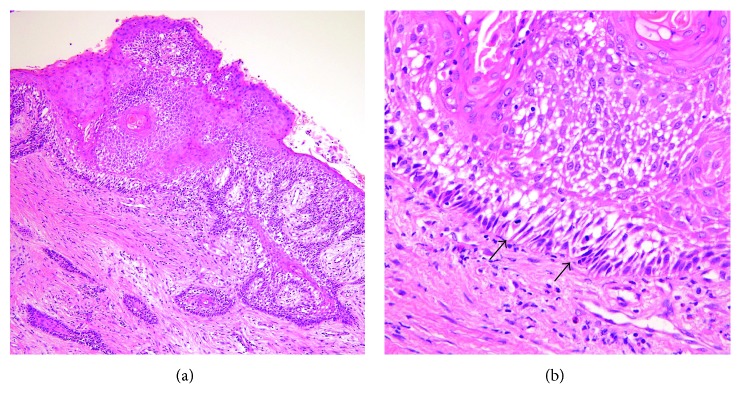
Unusual ameloblastomatous change of the OKC. (a) Only part of the lining epithelium shows papillary hyperplasia with ameloblastomatous epithelial elongation. (b) The suprabasal layer shows stellate reticulum-like formation, and the basal layer shows palisading tall columnar cell nuclei with focally mitoses (arrowheads) (HE; original magnification: ×40 (a) and ×200 (b)).

In January 2016, a 49-year-old Japanese woman visited a local dentist because of gingival swelling in the left upper canine area of the jaw, and a radiolucent lesion was found in the central maxilla. Although she had been aware of dull pain for five years, she had not taken any action because the symptoms improved. In July 2016, she was referred to Meikai University Hospital for detailed examination and treatment because of further gingival swelling and tooth displacement. Intraorally, the lesion involving the area from the upper left canine to the first premolar also showed evident buccal and palatal cortical expansion (Figures 1(a) and 1(b)). The epithelium overlying the mucosa was normal in color and unremarkable. Panoramic radiography showed an extensive, relatively well-delineated radiolucent lesion extending from the left middle incisor to the second premolar with tooth displacement but no root absorption (Figure 2(a)). A computerized tomography (CT) scan also demonstrated expansion and absorptive destruction of both the buccal and palatal cortical plates (Figures 2(b) and 2(c)). Clinically, the findings of a general examination were unremarkable. The clinical diagnosis was a suspected odontogenic tumor, and an incisional biopsy was performed. The histopathological appearance of a biopsy specimen was consistent with the odontogenic keratocyst (OKC) (Figures 3(a) and 3(b)). Surgery for the jaw tumor was performed intraorally under general anesthesia, and the tumor was resected along some peripheral bone tissues. Macroscopically, the surgical specimen was a cystic mass with a fibrous wall measuring 25 × 22 × 15 mm (Figure 4(a)). Microscopically, the lesion was cystic (Figure 4(b)) with a lining of parakeratinized stratified squamous epithelium. Although the pathological findings were almost consistent with the OKC, focally invasive atypical squamous cell epithelia ware noted (Figure 5(a)). Epithelial dysplasia was evident in the areas around the invasive atypical squamous cell nests (Figures 5(b) and 5(c)). Immunohistochemistry showed that the invasive atypical squamous cell nests (Figure 5(d)) were positive for p53 (Figure 5(e)) and that the proliferative activity (MIB 1 index) was about 20% (Figures 5(f)). Unusual ameloblastomatous epithelial elongation with a stellate reticulum was observed in part of the lining epithelium (Figure 6(a)) and also in the epithelium with mitoses (Figure 6(b)). Although postoperative blood tests demonstrated a high level of squamous cell carcinoma antigen (1.6 ng/mL), no tumor lesions were found in other organs by PET-CT and other radiological examinations. The postoperative histopathological diagnosis was most compatible with PIOSCC derived from the OKC.

**Figure 7:**
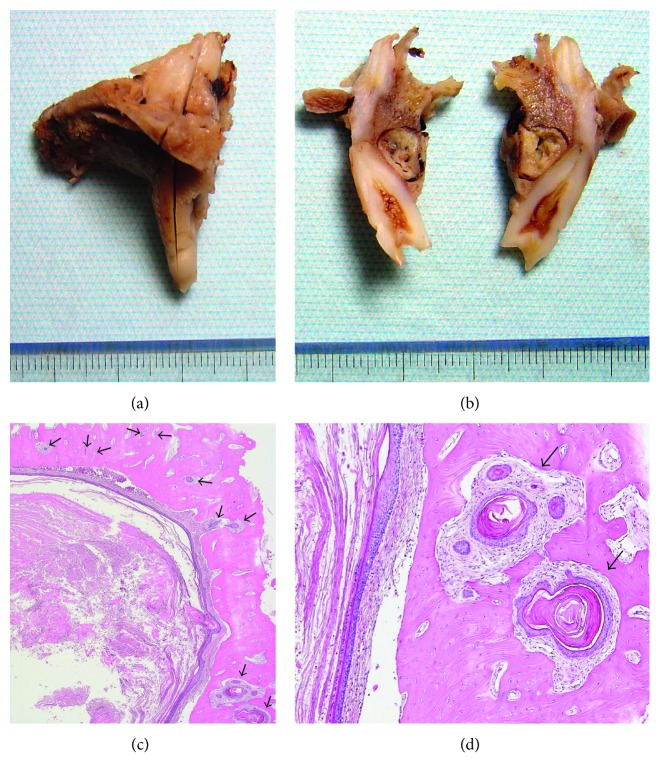
Macroscopic appearance and microscopic features of the additional surgically resected specimen. (a) Resected specimen. (b) Cystic lesion in the palatal side of the second premolar. (c) Microscopically, locally invasive daughter follicles in many bone marrow spaces (arrows). (d) Invasive solid cystic follicles with a KAB-like growth pattern (arrows) (HE; original magnification ×40 (c) and ×200 (d)).

Additional resection was performed because positivity at the distal surgical margin was suspected. This second surgical specimen measuring 35 × 30 × 25 mm included the left second premolar and maxillary sinus (Figure 7(a)). There was a cystic lesion, 8 × 7 mm in length, located in the palatal side of the second premolar (Figure 7(b)). Microscopically, this lesion appeared to be a typical OKC without malignant components, but there was marked local invasion to the bone marrow (Figure 7(c)). Solid variant OKC-like or keratoameloblastomatous microfollicles with keratin plugs were found in the bone marrow space (Figure 7(d)). The patient underwent additional resection, and the surgical margins were negative. No recurrence and distant metastasis were evident thereafter. The case study protocol was reviewed and approved by the Research Ethics Committee of the Meikai University School of Dentistry (A1321).

## 3. Discussion

Malignant changes in the epithelial lining of odontogenic cysts have been described previously [1]. Although the exact number of documented cases is difficult to determine, Gardner [5] reviewed all cases documented between 1889 and 1967 and considered 25 of them to be acceptable examples of malignant transformation of the epithelial lining of an odontogenic cyst. Gardner [5] proposed the following definitive criteria for identifying a lesion as PIOSCC derived from the odontogenic cyst: (i) a microscopic area of transition from a benign cystic epithelial lining to SCC, (ii) an intact overlying oral mucosa, (iii) the absence of carcinoma in adjacent structures, and (iv) the absence of metastatic carcinoma from a distant tumor [6]. Our present case fulfills all of these criteria. There are few documented examples of definitive histopathological transition between SCC and the epithelial lining of an odontogenic cyst [7]. In a review of 81 documented cases in the world literature, Woldron and Mustoe [6] considered the incidence of carcinoma arising from the odontogenic cyst to be approximately 1-2 per 1000 [8]. In a recent retrospective study of 116 cases of PIOSCC between 1938 and 2010, Bodner et al. in 2011 [3] found only 16 confirmed cases of PIOSCC arising from the OKC. PIOSCCs arising from OKCs are extremely rare, accounting for fewer than 30 cases reported so far [3, 4]. Evidence of a cystic component is a prerequisite for the diagnosis of PIOSCC arising from OKCs. The histopathologic criteria employed to document an odontogenic origin are (i) malignant transformation of the cyst lining, with a transition from the normal lining to dysplasia and to carcinoma, (ii) palisaded columnar cells, and (iii) the inductive influence of connective tissue [7]. All of these features were evident in the present case, and the most compatible diagnosis was PIOSCC derived from the OKC.

The pathogenesis of this lesion is still unknown. It has been reported that keratin metaplasia followed by hyperplasia and dysplasia of the cyst epithelium are signature events in the development of SCC in the OKC [9]. Also, the presence of keratinization in the cyst lining suggests a greater risk of malignant change [10]. Furthermore, Gardner [5] and Tamgadge et al. [11] have suggested that long-standing chronic inflammation is a factor related to malignant transformation of benign epithelium. In the present case, chronic inflammatory cell infiltration was seen in the fibrous connective tissue of the cyst wall (Figure 5(a)). The chronic inflammation in the present case was considered due to cyst epithelial damage resulting from degenerated keratins or cortical bone absorption resulting from growth and local invasion. Inflammation would likely occur as a stromal response to such tissue degeneration. In fact, inflammatory cell infiltration was also found in the cystic cavity (Figure 7(d)), and in routine pathological diagnosis, we often observed epidermal cysts with epithelial damage or an inflammatory reaction in response to keratin debris.

Unexpectedly, in the specimen obtained at initial surgery, unusual ameloblastomatous epithelial elongation was found in a limited part of the lesion, and in the second resected specimen, very small numerous invasive solid epithelial follicles composed of central lamellar para- or orthokeratin plugs were found in the bone marrow spaces on the outer side of the main cyst (Figures 7(c) and 7(d)). Histologically, these solid epithelial follicles had a keratoameloblastoma (KAB)/solid-OKC- (SOKC-) like growth pattern [12]. Ide et al. suggested that KAB might be derived from a preexisting OKC [13], and Geng et al. presented a supportive case in which the SOKC showed partial transformation to ameloblastomatous change [14]. About 40 years before (1977), Brannon [15] observed mural nodules of ameloblastoma as direct proliferations from the OKC lining, with an incidence of 0.6%. Therefore, we considered that the OKC might have the potential to differentiate into ameloblastoma. In the lining epithelium of odontogenic cysts, pseudoameloblastoma is often seen as a secondary morphological change induced by inflammation. In the additional resected specimen from the present case, no effect of inflammation was evident (Figures 7(c) and 7(d)). It is unknown whether true ameloblastomatous transformation had occurred, but the morphological appearance resembled KAB in part of this additional resected specimen. Therefore, we interpreted this by indicating that the OKC had been partially transformed to benign ameloblastomatous epithelium as associated anaplastic change. Accumulation of further case reports and in vitro studies of odontogenic cysts will be necessary to clarify the mechanism of epithelial differentiation and carcinogenesis caused by various factors including chronic inflammation. Our present case is considered to represent an extremely rare case of initial-stage PIOSCC derived from the OKC showing partial keratoameloblastomatous change in the maxilla.
